# Specification and DNA Barcoding of Thai Traditional Remedy for Chronic Kidney Disease: Pikad Tri-phol-sa-mut-than

**DOI:** 10.3390/plants10102023

**Published:** 2021-09-26

**Authors:** Suwimol Thariwong, Aekkhaluck Intharuksa, Panee Sirisa-ard, Wannaree Charoensup, Sunee Chansakaow

**Affiliations:** 1 Department of Pharmaceutical Sciences, Faculty of Pharmacy, Chiang Mai University, Chiang Mai 50200, Thailand; suwimol.t@psru.ac.th (S.T.); identity.int@gmail.com (A.I.); wannareecharoensup@gmail.com (W.C.); 2 Department of Integrated of Health, Aesthetics and Spa, Faculty of Science and Technology, Pibulsongkram Rajabhat University, Phitsanulok 65000, Thailand; 3 Faculty of Pharmacy, Chiang Mai University, Chiang Mai 50200, Thailand; pmpti008@gmail.com

**Keywords:** tri-phol-sa-mut-than, Thai traditional medicine, chronic kidney disease, *Aegle marmelos* (L.) Corrêa, *Coriandrum sativum* L., *Morinda citrifolia* L.

## Abstract

The Pikad Tri-phol-sa-mut-than (TS) remedy, a Thai traditional medicine, is officially recorded in Tamra Paetsart Sonkrau Chabub Anurak for its capabilities in treating kidney deficiency. TS remedy is composed of three fruit species—*Aegle marmelos* (L.) Corrêa., *Coriandrum sativum* L., and *Morinda citrifolia* L.—in an equal part by weight. The quality of the raw material is one of the essential factors that can affect the effectiveness and safety of treatment by herbal remedy. The pharmacognostic evaluation and DNA barcode of the three fruit species and TS remedy were performed in this study to authenticate them from contamination, and to provide the scientific database for further uses. Macroscopic and microscopic examination, chemical profile by TLC, and DNA barcoding were employed to positively identify the raw materials bought from the herbal market, especially the powder form. Consequently, the outcomes of this investigation can be used to develop an essential and effective tool for the authentication of crude drugs and herbal remedies.

## 1. Introduction

The remedy known as Pikad Tri-phol-sa-mut-than (TS) is a traditional form of medicine that has been apprised in Tamra Paetsart Sonkrau Chabub Anurak for its capabilities in treating kidney deficiency. TS remedy consists of *Aegle marmelos* (L.) Corrêa (Rutaceae), *Coriandrum sativum* L. (Apiaceae), and *Morinda citrifolia* L. (Rubiaceae) in equal weights [1]. Nowadays, the application of medications, kidney dialysis, and kidney transplants are considered to be high efficacy forms of treatment that are commonly administered to chronic kidney disease (CKD) patients. However, several side effects have been associated with some of the medications. CKD is a major risk factor for cardiovascular death in Thailand, while during the past two decades, incidences of end-stage renal disease (ESRD) have more than doubled in the country. The prevalence of stages 1–5 of CKD in Thailand, as has been reported in Thai Screening and Early Evaluation of Kidney Disease (Thai SEEK), was reported to be 17.5%. Importantly, this number has increased along with the age of the patient [2]. CKD is a worldwide public health issue that has been associated with poor outcomes and high costs due to the length of time required for treatment and the need for lifelong continuous medical care [3].

For thousands of years, traditional Chinese medicine (TCM) has been used to treat patients with CKD. Some of the herbal medicines administered in the CKD remedy are known to have been sourced from diuretic, antioxidant, and anti-inflammatory plants, while their activities have been effectively associated with kidney nourishment [4]. In contrast, there has been a lack of continuity in the use of Thai herbal preparations to treat CKD symptoms. A study on the toxicity and efficacy of traditional Thai medicine and/or folk medicine for CKD may provide new therapeutic options for patients. Thai traditional remedies may help improve CKD-related symptoms, improve the quality of life of patients, provide patients with a degree of protection, and delay the progress of the disease. The quality of the raw material is one of the factors that can influence the effectiveness of the treatment and the safety of the patients [5]. In this study, the pharmacognostic specifications of the three fruit species included in TS remedy were determined and assessed for use in preparing a set of guidelines for better quality control to be applied in future studies. In recent years, the identification of herbal medicinal materials by DNA barcode has also been widely applied to single herbs that have been modified for their use in medicinal remedies. The DNA barcodes can also be used as reliable tools for the authentication and quality control of herbal medicine or herbal remedies [6]. 

## 2. Results

### 2.1. Macroscopical and Microscopical Identification

**Figure 1:**
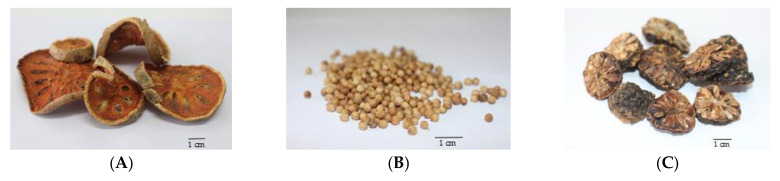
Macroscopic characteristics of crude drugs. (**A**) Fruits of *Aegle marmelos* (L.) Corrêa; (**B**) fruits of *Coriandrum sativum* L.; and (**C**) Fruits of *Morinda citrifolia* L.

**Figure 2:**
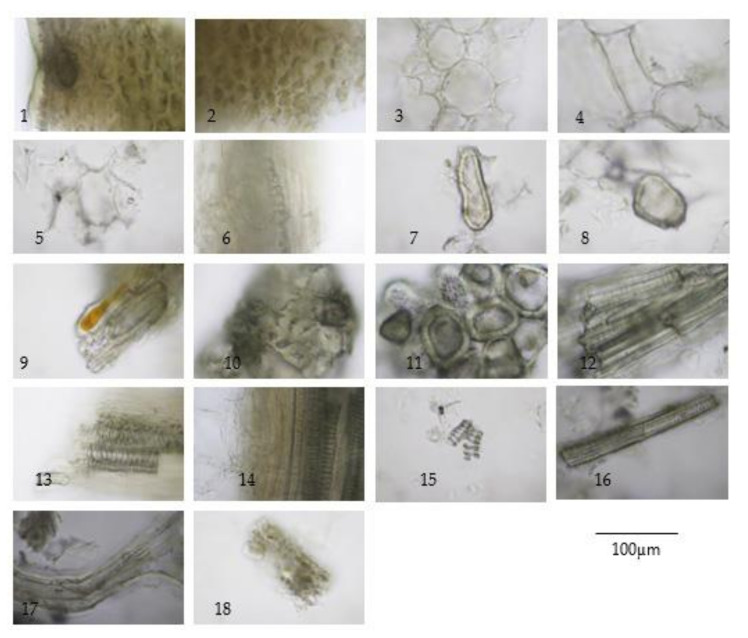
Diagnostic characteristics of *Aegle marmelos* (L.) Corrêa fruit powder: (**1**) epicarp in cross-sectional view showing cuticle layer, epidermis, thick-walled parenchyma, and lysogenous oil cavities; (**2**) thick-walled parenchyma from epicarp; (**3**) thick-walled parenchyma from mesocarp showing intercellular spaces; (**4**) thick-walled parenchyma in longitudinal view; (**5**) parenchyma containing prismatic crystals; (**6**) in-line prismatic crystals; (**7**,**8**) sclereids; (**9**–**11**) group of stone cells from mesocarp; (**12**) group of sclereids from endocarp; (**13**,**14**) vascular bundle from mesocarp; (**15**) spiral vessels; (**16**) pitted vessel; (**17**) seed hair; and (**18**) cotyledon in sectional view.

**Figure 3:**
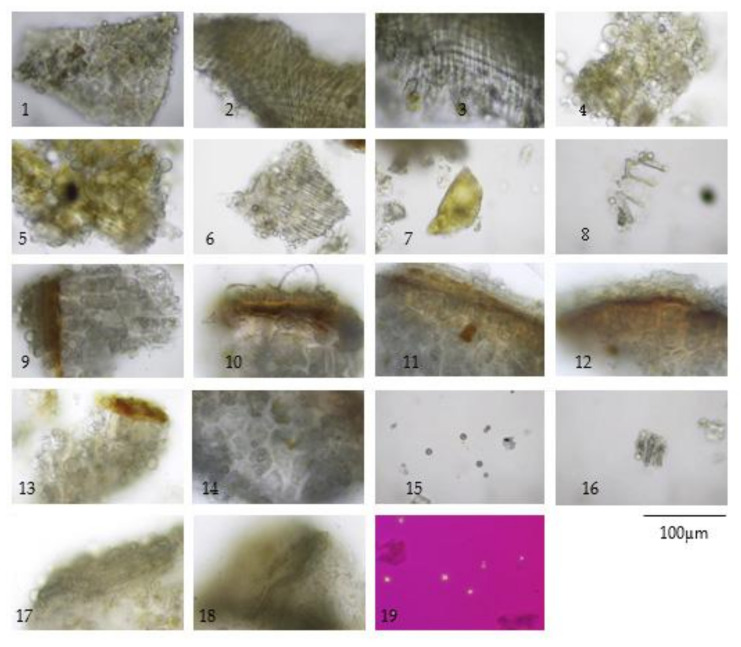
Diagnostic characteristics of Coriandrum sativum L. fruit powder: (**1**) epicarp in surface view; (**2**,**3**) group of fusiform sclereids of mesocarp showing two layers orientated at right angles to one another; (**4**) rectangular sclereids of mesocarp with underlying endocarp in surface view; (**5**) testa with underlying endocarp in surface view; (**6**) endocarp in surface view; (**7**) fragment of vitta (**8**) testa in surface view; (**9**,**10**) part of pericarp and seed in sectional view showing the endocarp, testa, and endosperm containing protein bodies, oil globules, and microspheroidal crystals of calcium oxalate; (**11**–**13**) seed in sectional view; (**14**) endosperm; (**15**) microspheroidal crystals of calcium oxalate; (**16**–**18**) vascular strand; and (**19**) microspheroidal crystals under polarized light.

**Figure 4:**
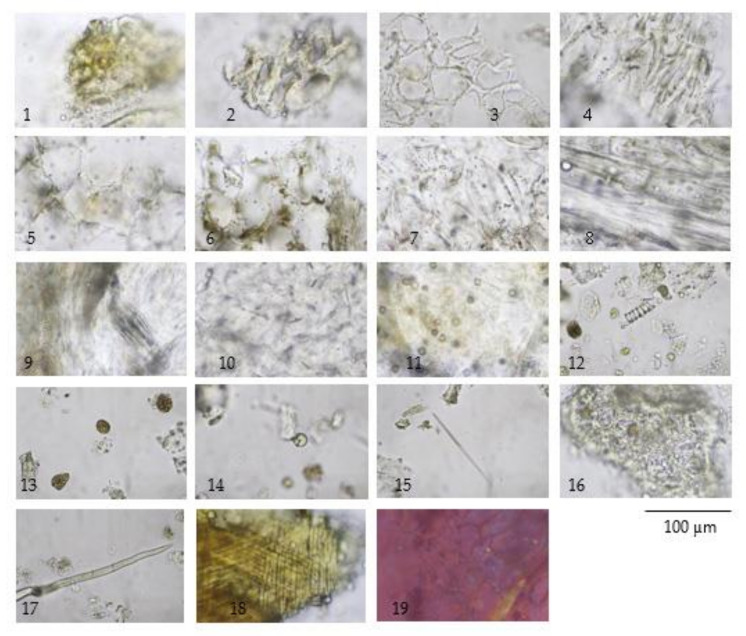
Diagnostic characteristics of *Morinda citrifolia* L. fruit powder: (**1**) epicarp; (**2**–**4**) thickening-walled parenchyma from fruit pulp in cross-sectional view (**2**,**3**) and longitudinal view (**4**); (**5**–**7**) thin-walled parenchyma in cross-sectional view (**5**,**6**) and longitudinal view (**7**); (**8**) thin-walled parenchyma associated with vascular strand (phloem tissue); (**9**) parenchyma with group of raphide and vascular strand; (**10**) parenchyma with scattered rod shape crystals; (**11**) parenchyma with microspheroidal crystals of calcium oxalate; (**12**) spiral vessel; (**13**) resin masses; (**14**) oil globules; (**15**) acicular crystal; (**16**) endosperm; (**17**) trichome; (**18**) fibrous layer of seed coat; and (**19**) microspheroidal crystals under polarized light.

**Figure 5:**
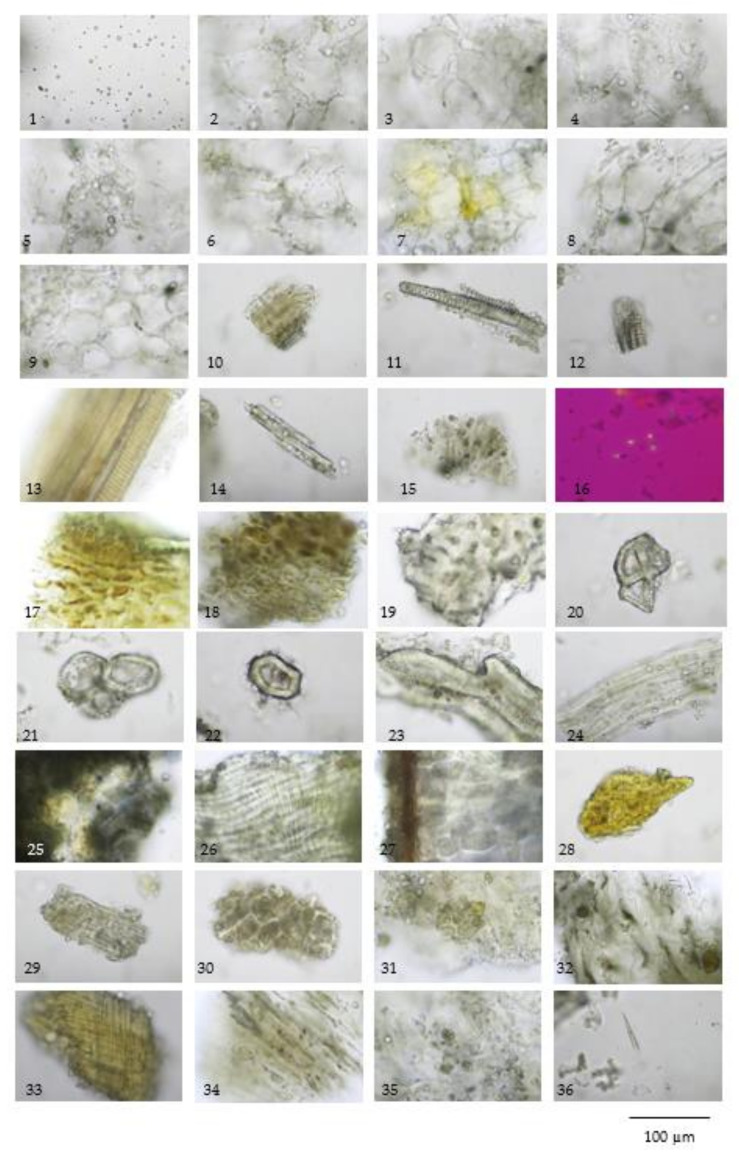
Diagnostic characteristics of Pikad Tri-phol-sa-mut-than (TS): (**1)** oil droplets; (**2**,**3**) thin-walled parenchyma; (**4**–**7**) parenchyma and oil droplets; (**8**,**9**) thick-walled parenchyma; (**10**–**13**) various size of vascular bundle; (**14**) isolated sclereids; (**15**) microspheroidal calcium oxalate crystals; (**16**–**24**) *Aegle marmelos* (L.) Corrêa fruit powder; (**16**) microspheroidal calcium oxalate crystals under polarized light; (**17**) epicarp in sectional view; (**18**) thick-walled parenchyma of epicarp; (**19**,**20**) group of sclereids from mesocarp; (**21**) group of sclereids from mesocarp; (**22**) isolated sclereid; (**23**) sclereids from endocarp; (**24**) seed hair; (**25**–**29**) *Coriandrum sativum* L. fruit powder; (**25**) epicarp in surface view; (**26**) group of fusiform sclereid of mesocarp; (**27**) seed in sectional view; (**28**) fragment of vittae; (**29**) endocarp in surface view; (**30**) endosperm; (**31**–**36**) *Morinda citrifolia* L. fruit powder; (**31**) epicarp; (**32**) thickening-walled parenchyma of fruit pulp; (**33**) fibrous layer of seed coat; (**34**) parenchyma with raphide; (**35**) protein bodies; and (**36**) acicular crystals.

The TS remedy is comprised of three fruit species in equal weights. The macroscopic and microscopic characteristics of these three species and the TS remedy itself are presented in Figure 1, Figure 2, Figure 3, Figure 4 and Figure 5.

A. The fruits of *A. marmelos* were dried. They appear round in shape or ovate, curved, 3–5 cm in width, and 0.5–1 cm thick. The rind appears greyish-brown to brownish, and it is smooth and hard. The pulp is shrunken, with a brownish orange or reddish brown color. It has radially arranged elliptical cavities, some of which contain seeds and a viscous substance.

B. The mature fruits of *C. sativum* were dried. They appear spherical in shape, and consist of two cremocarps that are 2–4 mm in diameter. Their color is yellowish-brown to brownish, and the surface is marked by 8–10 ridges. It is articulately-wrinkled, and hot to spicy in taste with an aromatic odor.

C. The fruits of *M. citrifolia* L. were dried. They appear circular in shape, 1.5–2.5 cm in diameter, and have 8–10 locule. They are brown to dark brown in color, and give off an unpleasant odor.

### 2.2. Physiochemical Identification

The physico-chemical examinations were as follows: total ash, acid-insoluble ash, water content, ethanol-soluble extractive value, water-soluble extractive value, and volatile oil content of the three fruits and TS remedy are presented in Table 1 along with their mean values.

### 2.3. Chemical Constituents Determined by TLC

**Figure 6:**
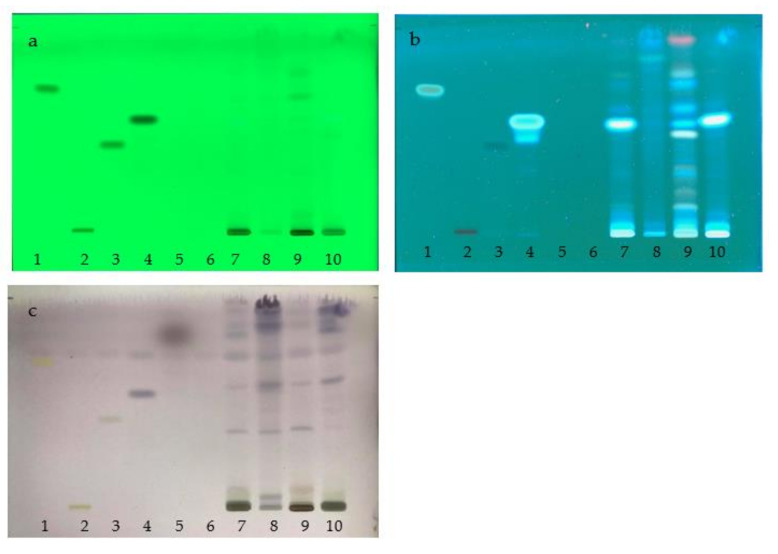
TLC chromatograms of water extract of TS remedy; (**a**) UV at 254 nm; (**b**) UV at 366 nm; (**c**) anisaldehyde-sulfuric acid spraying reagent; (1) kaempferol; (2) rutin; (3) gallic acid; (4) caffeic acid; (5) linalool; (6) cineol; (7) TS remedy; (8) *Coriandrum sativum* L. extract; (9) *Aegle marmelos* (L.) Corrêa extract; and (10) *Morinda citrifolia* L. extract.

**Figure 7:**
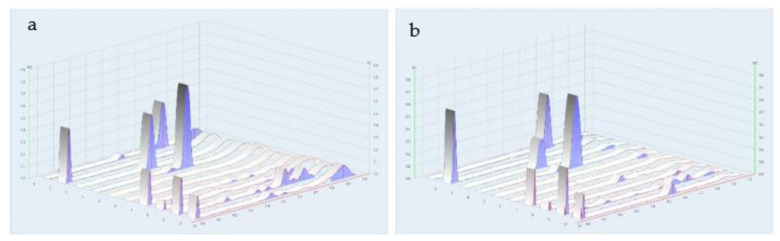
Densitograms of water extract of TS remedy. (**a**) UV at 254 nm; (**b**) UV at 366 nm; (1) kaempferol; (2) rutin; (3) gallic acid; (4) caffeic acid; (5) linalool; (6) cineol; (7) TS remedy; (8) *Coriandrum sativum* L. extract; (9) *Aegle marmelos* (L.) Corrêa extract; and (10) *Morinda citrifolia* L. extract.

The chemical profiles of the water extracts of A. *marmelos* (fruit), *C. sativum* (fruit), *M. citrifolia* (fruit), and the TS remedy were determined by TLC with the use of a detector under UV light at 254 and 366 nm. The plate was sprayed with a freshly prepared anisaldehyde-sulfuric acid reagent or natural product spraying reagent (Figure 6 and Figure 7). The chemical constituents found in TS included a mixture of these three fruit species. The major component, Rf 0.55, originated from the fruits of *A. marmelos* and *M. citrifolia*. The minor components were found in amounts of Rf 0.06, 0.1, 0.3, 0.62, 0.73, 0.76, and 0.91 as the compounds present in *A. marmelos*, *C. sativum*, and *M. citrifolia*. Comparative to chemical standards, the TS remedy found caffeic acid (Rf 0.55) as a principal component. Linalool (Rf 0.82), kaempferol (Rf 0.73), and gallic acid (Rf 0.43) were revealed as the minor components. (Figure 6 and Figure 7).

### 2.4. DNA Barcoding Analysis 

**Figure 8:**
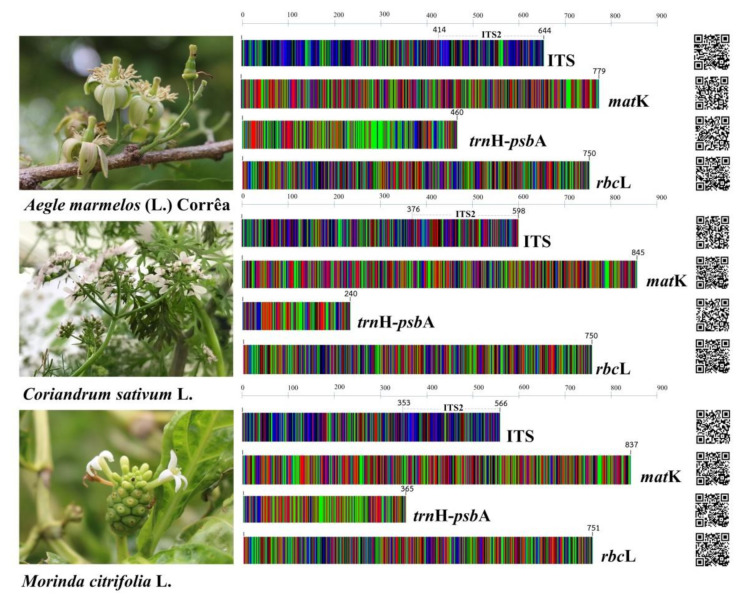
DNA barcoding images of four DNA barcode loci: ITS, *mat*K, *psb*A-*trn*H, and *rbc*L belonging to *Aegle marmelos* (L.) Corrêa, *Coriandrum sativum* L., and *Morinda citrifolia* L., respectively. Different colors are representative of different bases: green base A, red base T, blue base C, and black base G. The quick response code (QR code) for accession to nucleotide sequences was submitted to DDBJ/ENA/GenBank genetic database.

We successfully amplified and sequenced the DNA barcode of all of the core barcode loci for 22 samples of these three species, which were used as ingredients in the TS formulation. All nucleotide sequences were deposited in the DDBJ/ENA/GenBank genetic database under accession number LC633819-633830, as is shown in Table 2. These sequences can be promptly accessed via the quick response code (QR code) presented in Figure 8. In order to analyze the nucleotide sequences, the nuclear ITS and ITS2 intergenic sequences that were part of the ITS region together with the plastid regions of *mat*K, *trn*H-*psb*A, and *rbc*L, were assessed (Figures S1–S5). The length ranges of the consensus DNA fragments of ITS2, ITS, *mat*K, *trn*H-*psb*A, and *rbc*L were 212–230 bp, 566–645 bp, 779–845 bp, 240–460 bp, and 750–751 bp, respectively (Figure 8). We found that there are different lengths of the nucleotide sequences in all DNA barcode loci among these three species. For example, in the *trn*H-*psb*A region, the shortest length was found in *C. sativum* (240 bp), followed by *M. citrifolia* (365 bp) and *A. marmelos* (365 bp). Therefore, they were enabled to discriminate using the different length of amplicons. Moreover, the lowest percentage of the Guanine and Cytosine base was *trn*H-*psb*A, followed by *mat*K, *rbc*L, ITS, and ITS2. Three species were collected from different areas, but the nucleotide sequences of all regions were found to be identical. Furthermore, the intraspecific variations were zero. An analysis of the pairwise genetic distance between the three species using the K2P model of evolution was conducted. We found that the highest average of the overall variations was 0.536 ± 0.05 in *trn*H-*psb*A, followed by 0.522 ± 0.06 in ITS2, 0.381 ± 0.03 in ITS, 0.263 ± 0.02 in *mat*K, and 0.091 ± 0.01 in *rbc*L (Table 3).

### 2.5. Generation of the Predicted ITS2 Secondary Structure

**Figure 9:**
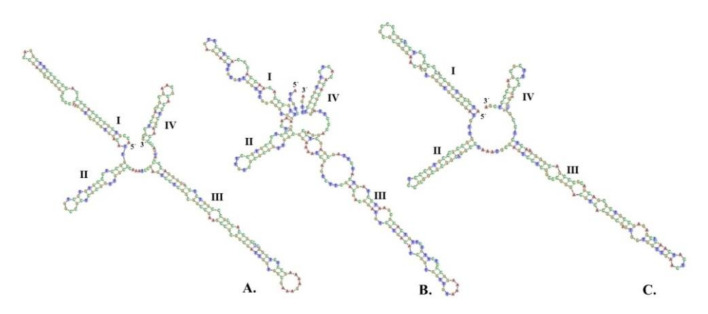
Predicted ITS2 secondary structure using Homology Modelling (HM) of (**A**) *Aegle marmelos* (L.) Corrêa; (**B**) *Coriander sativum* L.; and (**C**) *Morinda citrifolia* L. as ingredients of the TS remedy.

According to the result of the interspecific K2P genetic distance, *trn*H-*psb*A and ITS2 were selected for discriminating between three species in TS formulation. Because the ITS2 secondary structure contains a high conserve of nucleotide motifs which anchor the multiple sequence alignment, the secondary structure of ITS2 presented a folding shape, with four helices exhibits that were more realistic and clearly visible than the *trn*H-*psb*A secondary structure presented in a hairpin shape [7,8]. Thus, the patterns of the ITS2 secondary structures were predicted for identifying any discrimination among the three species. As a result, we found that all of the ITS2 secondary structures exhibited the same pattern, which consisted of four helicoidal ring models: helix I, II, III, and IV radiating around the central ring. This has been illustrated in Figure 9. The percentages of helical transferring parameters of four helices (I/II/III/IV) achieved from homology modeling were 90.90/81.81/96.77/80.00 in *A. marmelos*, 93.75/100/100/100 in *C. sativum*, and 90.48/100/100/75 in *M. citrifolia*. The resulting ITS2 secondary structure of *C. sativum* (Figure 9B) was different from those of the *A. marmelos* (Figure 9A) and *M. citrifolia* (Figure 9C) in terms of the corresponding position, quantity, and size of the loop in helix III, along with the asymmetry of the central ring. Furthermore, the secondary structure between *A. marmelos* and *M. citrifolia* was established with regard to the size of the central ring and the morphological characteristics of the four helices.

### 2.6. *Authentication of Crude Drugs* of Aegle marmelos *(L.)*, Coriandrum sativum *L.* Corrêa, *and* Morinda citrifolia *L.*

According to the nucleotide sequence analysis, the plastid *trn*H-*psb*A and ITS2 region exhibited a high interspecific variation among the three species. Therefore, they were determined to be an appropriate DNA locus for authenticating the botanical origin of the three crude drugs. In this study, we ultimately achieved the amplification of the PCR products of the *trn*H-*psb*A and ITS2 intergenic region in all expected crude drugs. All PCR products were then analyzed in terms of the nucleotide sequences of both DNA loci. The obtained DNA sequences of the three crude drugs were compared to the reference nucleotide sequences of the authentic samples. All sequences were revealed to be utterly similar. In addition, the DNA sequences of the ITS2 region were used to construct the secondary structures. The results manifested the concomitant size and number of the helices and loops of the secondary structure between the authentic species and the expected crude drugs.

## 3. Discussion

Presently, it has been conceded that chronic kidney disease is increasingly becoming a global public health problem. This trend has also been observed in Thailand. Accordingly, Thai traditional remedies for CKD-related symptoms have been recorded in the traditional Thai medicine manuscript and the National List of Essential Medicines of Thailand, both of which have received official approval from the Thai Ministry of Public Health. However, there has been a lack of continuity with regard to the longstanding use of these herbal remedies [9]. At present, authoritative information on the use, safety, and efficacy of these remedies has been limited. Nevertheless, it has been noted that some of the herbal medicines in TTM can delay the progress of CKD or improve the quality of life of the patients who use them. Notably, some herbal medicines may even act as nephroprotective agents [10]. Therefore, Thai traditional medicine may provide alternative therapeutic options for patients who suffer from chronic kidney disease. A surge in the popularity and usage of these herbal medicines has been augmented worldwide. However, quality irregularities have been reported in the raw materials and finished products of some of these herbal medicines. It has been continuously realized that these irregularities can directly affect the efficacy and safety of these medicines [11]. The multidisciplinary scientific approaches described in various official publications, namely viz Pharmacopoeia and WHO Monographs on Medicinal Plants, can be applied to raw plant materials, extracts, and finished products for the purposes of quality control. In the herbal industrial process, raw plant materials are utilized at the initial stage of the core processing steps along the herbal product pipeline [12]. The identification and authentication of herbal drug substances are necessary and inevitable processes that ensure the authenticity of the originating materials before further steps can be taken. The results of organoleptic, macroscopic, and microscopic analyses, as well as a chemical analytical assessment of the raw plant materials, were referenced in the Monograph of Pharmacopoeias to determine the botanical origins of the crude drugs. Moreover, molecular analysis has been successfully used in the species-level identification of several herbal medicines over the latest decade [13,14,15]. 

The three crude drugs contained in the TS remedy were easily identified based on plant morphology, as is shown in Figure 1. However, the identification process can become even more complicated when the raw material is in powder form and purchased from a local herbal drug store. The powder form of the TS remedy contains oil droplets and ground parenchyma (thin-walled parenchyma displaying intercellular spaces) (Figure 5 (1–7)), vascular bundle (Figure 5 (10–13)) isolated sclereids (Figure 5 (14)), and microspheroidal calcium oxalate crystals (Figure 5 (15–16)), of which are probably from *M. citrifolia* and/or *C. sativum* that commonly appear. The specific parenchyma (Figure 5 (24)), which is narrowly elongated in 7–12 cell-thick segments has been associated with scattered oil droplets that represent the seed hairs of *A. marmelos* seeds. Notably, the vascular tissue containing xylem elements with spiral and rarely pitted vessels have been infrequently observed (Figure 5 (10–13)). The very abundant thick-walled parenchyma usually appear unspecified (Figure 5 (8–9)), but some contain chloroplasts that originate from the epicarp of the *A. marmelos* fruit (Figure 5 (17–18)). A thick-walled parenchyma is of a larger size and possesses a wavy wall that can be classified with pulp of *M. citrifolia* (Figure 5 (32)). The fair sclereid obtained from the mesocarp of *A. marmelos* is usually identified as macrosclereid (groups or solitary) rather than brachysclereid (large groups of compressed, polygonal, thick-walled sclereids and smaller lumens) (Figure 5 (19–22)). The fusiform sclerenchyma appear with narrow lumens, which were present in several layers, along with the orientation of the cells at right angles to one another, and were moderately found (Figure 5 (26,32)). The sclereids of the mesocarp of *C. sativum* or the seed coat of *M. citrifolia* were plainly observed. These two sclereids could be differentiated from each other by their colorless and yellowish appearances, respectively. The moderately found seeds of *C. sativum* in the sectional view were distinctly recognized by a brownish single layer of the testa and endosperm cells, which contained microspheroidal crystals of calcium oxalate (Figure 5 (27,30)). In terms of the acicular crystals, the protein bodies obtained from the *M. citrifolia* and endocarp of *C. sativum* were seldomly observed (Figure 5 (29,35,36)). A remarkable feature of the microscopic characteristics of the TS remedy was derived from the high-frequency tissues found in the raw materials, which were also used to identify the dominant features manifested in the plant tissue (Supplementary data S6).

According to a physico-chemical examination, total ash, acid-insoluble ash, ethanol-soluble extractive value, water-soluble extractive value, water content, and volatile oil content of the crude drugs were used for quality evaluations. The three plant species are well-known herbs, but only the fruit of *A. marmelos* was officially established in THP 2018. Therefore, the physico-chemical data of *C. sativum* (fruit) and *M. citrifolia* (fruit) were compared with that of the published reports on plant specifications [16,17]. Physico-chemical examinations of these two species were performed following the methods established in THP 2018. As a result, the quality of *A. marmelos* was accepted according to THP 2018. Specifications of the crude drugs of *C. sativum* and *M. citrifolia* will support the quality control of raw materials in further studies [18]. Discrepancies in some values observed in our physico-chemical examination, along with some that were recorded in a previous report, may have been due to certain differences in the geographical, climatic, post-harvesting process, and/or experimental conditions. Physico-chemical examination of the polyherbal TS remedy was the average constant value from the mixture of three fruit species (Table 1). These data will be one of the criteria to evaluate the quality of TS remedy for further study.

The chemical profiles of the water extract of *A. marmelos*, *C. sativum*, *M. citrifolia*, and the TS remedy were evaluated by TLC with detection under UV light at 254 (Figure 1a) and 366 nm (Figure 1b). Furthermore, an anisaldehyde-sulfuric acid spraying reagent (Figure 1c) was applied. Caffeic acid (Rf 0.55), originating from the fruits of *A. marmelos* and *M. citrifolia*, was found to be a principal component of the fruits (Figure 6 and Figure 7). Linalool (Rf 0.82), kaempferol (Rf 0.73), and gallic acid (Rf 0.43) were revealed as the minor components in the chemical profile of the TS remedy (Figure 6 and Figure 7). The chemical constituents present in TS were comprised of a mixture of these three species [19,20,21,22,23,24,25,26,27,28,29,30]. The chemical profiles established by the chromatographic techniques can be beneficial for quality control and in determining the authenticity of raw materials and herbal products. The specifications of the herbal remedies reported in this study can be used as a set of guidelines for quality control and as a point of reference in further research on the repeatability of the extract preparation process.

DNA barcoding, a short standard DNA region utilized for species-level identification of living organisms, has been broadly applied in areas of taxonomy, the trade in flora and fauna, and forensic sciences. DNA barcoding can also be applied in the preparation processes of foods and medicines [12]. In this study, a DNA barcoding analysis was performed to establish the reference nucleotide sequences and to authenticate the crude drugs that are mixed in the TS formulation. The nuclear ribosomal RNA gene ITS region—including the ITS2 intergenic area and three plastid regions, namely *mat*K, *trn*H-*psb*A, and *rbc*L as a core barcode—were analyzed to determine the discriminatory performance among the three species. Based on the pairwise genetic distance analysis assessment, *trn*H-*psb*A and ITS2 exhibited a high interspecific degree of divergence (0.536 ± 0.05 and 0.522 ± 0.06, respectively) among the five core barcode loci. Meanwhile, the other DNA barcode regions presented a lower interspecific genetic distance among three species. However, the ingredients composed of the TS formulation belong to different genus, so the nucleotide sequences in all core barcodes enable to distinguish among these three species. As a result, *trn*H-*psb*A and ITS2 exhibited a high discriminatory potential, and could be considered helpful as a barcode for identifying or authenticating plants at the species level. This determination is consistent with the findings of previously published research works [31,32,33,34,35,36]. Among the three fruit species analyzed in this research, we found that the length of the nucleotide sequences of the *trn*H-*psb*A region were different. As a result, the amplicons of trnH-psbA enabled us to achieve the easy detection of herbal medicine [37]. The ITS2 region has overwhelmed the difficulties associated with the amplification process in the highly degraded DNA of many herbal medicines. This is because they were found to contain short sequences (approximately 200–230 bp) [13,38,39]. Therefore, ITS2 was identified as a good barcode for the purposes of identification and authentication of herbal medicines used as raw materials, processed materials, and finished products [39,40,41,42]. Additionally, many research articles have reported the need to integrate trnH-psbA and ITS2 regions for plant species identification [43,44,45,46]. In order to establish more convenient and rapid identification processes, the ITS2 secondary structures of these three species were predicted. Among the three species, their secondary structures demonstrated apparently different helices with regard to the number of stem loop, size, position, and characteristics of the central ring. It has been stated that the secondary structure of ITS2 can be useful for species identification at the molecular morphological characteristic level. This structure can be applied to identify various herbal medicines such as *Ligusticum sinense* (Apiaceae) [47]; *Rhodiola crenulata* and *R. rosa* (Crassulacea*e*) [48]; *Trachelospermum jasminoides* (Apocynaceae) [49], the Chinese medicinal plant in Apocynaceae [46]; and various other ingredients of the traditional Chinese herbal remedy “Mu Tong” [7]. 

## 4. Materials and Methods

### 4.1. Plant Materials

#### 4.1.1. Collection of Crude Drugs for Specification Evaluation 

Plant materials were purchased from local crude drug markets in Thailand. All plant samples were identified by comparison with the authentic samples listed in a botanical herbarium or described in official manuscripts/monographs. The samples were then cleaned, reduced in size, and dried at 50 °C in a hot air oven.

#### 4.1.2. Collection of Plant Materials for DNA Barcoding Analysis

Twenty-two species of *Aegle marmelos* (L.) Corrêa (Rutaceae), *Coriandrum sativum* L. (Apiaceae), and *Morinda citrifolia* L. (Rubiaceae) were collected from areas located in the central, north-eastern, and the northern part of Thailand (Table 4). Mature leaves of individual specimens were kept in a plastic bag with silica gel for further study. All voucher samples were identified by Wannaree Charoensup, a botanist at the Faculty of Pharmacy, Chiang Mai University, and submitted to the Herbarium of the Faculty of Pharmacy, Chiang Mai University (CMU) in Chiang Mai, Thailand.

### 4.2. Preparation of Crude Extracts 

Each plant material was reduced in size, pulverized, and formulated according to the ratios described in the manuscript. The extraction process was conducted according to traditional methods (samples were soaked in water and boiled, then the temperature was reduced and simmered until concentrated). The coarse powder was extracted over a period of 30 min, wherein water was used as a solvent. The extract was filtrated, concentrated to %brix = 3, and then dried using a spray dryer. The conditions of the spray drier were as follows: an inlet temperature of 140 °C, an outlet temperate of 80 °C, an aspirator flow value of 100%, a point of pump value of 30%, and the nozzle cleaner was set to a 2 sec interval.

### 4.3. Specification of TS Recipe

The formulation for this remedy was assessed in terms of its pharmacognostic and physico-chemical properties following the official methods explained in Thai Herbal Pharmacopoeia 2018 (THP2018). 

#### 4.3.1. Determination of Macroscopic and Microscopic Characteristics 

Plant materials were categorized according to their sensory macroscopic and microscopic characteristics. The procedure of identification involved three main steps: the selection of typical materials, the preparation of slides or powder, and the observation of relevant features.

Photomicroscope, the microscope evaluation, was commonly conducted using a digital camera attached to the microscope. The photographs were recorded with an attached digital camera (Canon EOS 760D) and examined under the photomicroscope (Nikon Eclipse E200) using an appropriate objective lens and eyepiece lens. The images were recorded using the EOS Utility 3 program.

#### 4.3.2. Determination of Loss on Drying

Powdered drugs were weighed out at 2–5× *g* and put into weighing bottles. The test samples were dried in a hot air oven at 105 °C until a constant weight was achieved. They were then cooled in a desiccator. The loss in weight was recorded as a determination of moisture content.

#### 4.3.3. Determination of Water Content 

About 10× *g* of the powdered drug was weighed and put into a distilling flask. Water-saturated toluene at a volume of 200 mL was added, and the solution was distilled using an azeotropic apparatus until the water was entirely removed. The water layer in the receiving tube was then evaluated, and the content of the water was calculated as a percentage of the dried material.

#### 4.3.4. Determination of Ash Values

Determination of Total Ash

About 2–3× *g* of the powdered drug was then weighed and put into a completely dried and weighed crucible. The test sample was ignited gradually in an electrical muffle furnace (Thermo Fisher, Massachusetts, USA), and the heat was increased to 500 °C until it was determined to be carbon-free. The test sample was then cooled in a desiccator and reweighed.

Determination of Acid-Insoluble Ash

About 25 mL of 2 M HCl was added to the crucible containing the total ash. The crucible was covered with a watch-glass and then gently boiled for 5 min in a water bath. The insoluble matter was filtered with No. 41 filter paper and washed with hot water until the filtrate was neutral. The filter paper containing the insoluble matter was transferred to the crucible and ignited gradually in an electrical muffle furnace to achieve a constant weight. The test samples were then cooled in desiccators and weighed. 

#### 4.3.5. Determination of Extractive Value

Ethanol Soluble Extractive Value

Initially, 5.0× *g* of the powdered drug was transferred to a glass-stopper conical flask, macerated with 100.0 mL of 95% ethanol for 6 h, frequently shaken, and then allowed to stand for 18 h. The sample was filtered and the marc was washed with ethanol to adjust the filtrate to 100.0 mL. Subsequently, 20.0 mL of the filtrate was transferred to a pre-weighed evaporating dish. The test sample was then evaporated to dryness in a water-bath and dried at 105 °C, cooled in a desiccator, and weighed.

Water-Soluble Extractive Value

Approximately 5.0× *g* of the powdered drug was macerated with 100.0 mL of water under shaking for 6 h and then allowed to stand for 18 h. The sample was filtered, the marc was washed with water to adjust the filtrate to 100.0 mL, and 20.0 mL of the filtrate was then transferred to a pre-weighed evaporating dish. The test sample was eventually evaporated to dryness in a water-bath, dried at 105 °C, cooled in a desiccator, and weighed.

#### 4.3.6. Determination of Volatile Oil

Approximately 10.0× *g* of the powdered drug was weighed and transferred to a round-bottomed flask. Subsequently, 100 mL of water was then added as a solvent, and the specimen was distilled for 5 h. The level of the essential oil was read as a volume in milliliters, and the percentage of the volatile oil content in the sample was calculated. 

#### 4.3.7. Thin Layer Chromatographic Fingerprint 

Plates at a thickness of 0.25 mm were precoated with TLC Merck® Silica gel 60 F254 and used in the experiments. The detection of spots was achieved via irradiation at UV (254 nm and 366 nm) and with the use of chemical reagent (anisaldehyde-sulfuric acid) followed by heating. Information provided by the finished chromatogram indicated the migrating behavior of the separated substances that were evaluated in the form of an Rf value.

Mobile phase (condition 1)—Toluene: Ethyl acetate: Formic acid (5:4:1)

#### 4.3.8. DNA Extraction, Amplification, and Sequencing

Mature leaf specimens were desiccated in silica gel and pulverized into a fine powder using a mortar and pestle in conjunction with liquid nitrogen. Individual samples were extracted using a DNeasy Plant Mini Kit™ (Qiagen, Hilden Germany), following the manufacturer’s instruction with minor modifications. The quality and quantity of the obtained genomic DNA was evaluated using a NanoDrop One UV-Vis spectrophotometer (Thermo Scientific, Massachusetts, USA) and agarose gel electrophoresis. Total genomic DNA was kept at −20 °C for further analysis. In this study, we amplified the nuclear ribosomal internal transcribed spacer (ITS) and three plastid regions—namely maturase K (*mat*K), ribulose-1.5-bisphosphate carboxylase/oxygenase large subunit (*rbc*L), and *trn*H-*psb*A intergenic spacer—to serve as representatives of the core DNA barcode region. Accordingly, we followed the procedure described in previous studies [50,51,52]. The universal primers of all DNA barcode loci are shown in Table 5. A volume of 25 µL of the PCR reaction was comprised of 2X GoTaq® Green Master Mix (Promega, WI, USA) at 12.5 µL, 10 µM of each of the forward and reverse primers at 1 µL, a DNA template of approximately 200 ng, and nuclease-free water. Simultaneously, a negative control as a reaction mixture without genomic DNA was taken at the PCR step. The PCR conditions for all of the core DNA barcode loci were as follows: 95 °C for 5 min for denaturation; 40 cycles at 95 °C for 1 min followed by 45 °C for 1 min for ITS and *trn*H-*psb*A loci; and 72 °C at 2 min with a final extension at 72 °C for 10 min. The aligned PCR products were electrophoresed by 1.8% agarose gel stained with RedSafe™ (Intron Biotechnology, Gyeonggi, Korea) and detected using Gel Doc™ EZ Imager (Bio-Rad, CA, USA). The successful PCR amplicons were purified using a MEGAquick-spinTM Plus Total Fragment DNA Purification Kit (Intron Biotechnology, Gyeonggi, Korea), and subsequently bidirectionally sequenced using a ABI PRISM 3730 XL sequencer (Applied Biosystems, MA, USA). The obtained nucleotide sequences were then manually corrected and trimmed, and the primer regions and low-quality regions were removed using BioEdit software version 7.2.6 [53]. DNA sequences were aligned using MUSCLE software [54] and MEGA software version X [55]. Each corrected nucleotide sequence confirmed the species using the Basic Local Alignment Search (BLAST) (https://blast.ncbi.nlm.nih.gov/Blast.cgi) 13 March 2021. All sequences were submitted to the DDBJ/ENA/GenBank genetic sequence database, and accession numbers are shown in Table 2. Sequence length and the average values of the G and C bases of all DNA barcodes were analyzed. Furthermore, intraspecific and interspecific divergence values were determined using the Kimura-2 parameter model of evolution with 10,000 boot-strap replications [56].

#### 4.3.9. Prediction of ITS2 Secondary Structure

The accurate boundary of the ITS2 sequence was determined based on the Hidden Markov models (HMMs) [64]. Subsequently, the secondary structure of ITS2 was determined using Homology Modelling (HM) of the ITS2 (Internal Transcribed Spacer 2 Ribosomal RNA Database) Workbench (http://its2.bioapps.biozentrum.uni-wuerzburg.de) access date 18 May 2021, with a default setting at 75% threshold for helix transfer similarity. A gap open penalty of 20 and a gap extension penalty of 4 were used for the transfer helices [65,66].

#### 4.3.10. Authentication of the Ingredients of TS Formulation

The botanical origins of the ingredients used in the TS recipes, namely Matum, Look Phak Chi, and Yo, as *Aegle marmelos*, *Coriandrum sativum,* and *Morinda citrifolia*, respectively, were confirmed. Powdered samples of the three crude drugs were extracted to assess the genomic DNA using the same method that was employed with the leaf samples, while the *trn*H-*psb*A and ITS2 regions were selected to be amplified for the purposes of authentication of the crude drugs. For the t*rn*H-*psb*A region, *trn*H-*psb*AF/*trn*H-*psb*AR was utilized as a primer set for the crude drug substances of Matum and Look Phak Chi, while Yo was used as the primer set for MOC-*psb*AF/MOC-*psb*AR (Table 2). In the ITS2 region, a primer set for the Un3F/Akebi-26SR ITS2 region (Table 2) was utilized in all of the crude drugs. A PCR reaction was carried out with the resemble protocol of leaf specimens. PCR amplification was then carried out under the following conditions: 96 °C for 5 min, 40 cycles at 95 °C for 1 min, 45 °C for 1 min, 72 °C for 2 min, and 72 °C for 10 min. The amplified products were then visualized by electrophoresis on 1.8% (*w*/*v*) agarose gel and purified using a MEGAquick-spinTM Plus Total Fragment DNA Purification Kit (Intron Biotechnology, Gyeonggi, Korea). The purified PCR products were directly sequenced using a sanger sequencer. The raw data of the nucleotide sequences were edited and ultimately aligned. Subsequently, BLAST analysis was employed to analyze the specimens using the nucleotide database at the National Center for Biotechnology Information (NCBI) (https://blast.ncbi.nlm.nih.gov/Blast.cgi) 31 May 2021. In addition, the obtained nucleotide sequences of each crude drug were compared with the nucleotide sequences of the reference samples, while the secondary structure of the ITS2 sequence was predicted following the above-mentioned protocol.

## 5. Conclusions

Macroscopic and microscopic examination, chemical profile by chromatographic technique, and DNA barcoding were employed to positively identify the raw materials used in the form of crude drugs or powder. Physico-chemical investigations and chemical fingerprints can be used to develop a set of guidelines for the quality control of raw materials and herbal products. Consequently, the outcomes of this investigation can be used to develop an important and effective tool for the authentication of crude drugs and herbal remedies.

## Figures and Tables

**Table 1:** Pharmacognostic characteristics of three fruit species and TS remedy.

Specification	Content (%)			
	AM	CS	MC	TS
Loss on drying	8.42 ± 0.02	-	-	-
Total ash	3.95 ± 0.07	8.18 ± 0.05	6.77 ± 0.04	6.55 ± 0.03
Acid-insoluble ash	0.07 ± 0.02	0.06 ± 0.02	0.10 ± 0.01	0.07 ± 0.00
Ethanol-soluble extractive value	11.66 ± 0.04	17.19 ± 0.17	27.33 ± 0.13	21.75 ± 0.08
Water-soluble extractive value	48.95 ± 0.09	17.21 ± 0.24	48.95 ± 0.14	37.69 ± 0.21
Water content	-	4.33 ± 0.29	4.50 ± 0.00	5.50 ± 0.00
Volatile oil content	-	1.50 ± 0.00	-	0.50 ± 0.00
Each sample analysis was performed in triplicate.				

AM—*Aegle marmelos* (L.) Corrêa; CS—*Coriandrum sativum* L.; MC—*Morinda citrifolia* L.

**Table 2:** List of collected samples for generation of core DNA barcodes of the compositions in the TS formulation.

Composition of TS Formulation	Voucher Numbers	Locality (District, Province)	Accession Numbers			
			ITS	*mat*K	*trn*H-*psb*A	*rbc*L
*Aegle marmelos* (L.) Corrêa	Authentic Specimens					
	AEM-PL06032021	Mueang, Phitsanulok				
	AEM-CM11032021	Mueang, Chiang Mai	LC633819	LC633825	LC633828	LC633822
	AEM-AC24032021	Mueang, Amnat Charoen				
	AEM-UR24032021	Det Udom, Ubon Ratchathani				
	AEM-KP28032021	Mueang, Kamphaeng Phet				
	AEM-PL29032021	Phrom Phiram, Phitsanulok				
	AEM-PB30032021	Prachantakham, Prachinburi				
	AEM-KP31032021	Phran Kratai, Kamphaeng Phet				
	Crude drugs					
	MAT-NP08082018	Mueang, Nakhon Pathom	-	-	-	-
*Coriandrum sativum* L.	Authentic specimens					
	COS-KP06032021	Mueang, Kamphaeng Phet				
	COS-KP08032021	Mueang, Kamphaeng Phet				
	COS-CM11032021	Mueang, Chiang Mai	LC633820	LC633826	LC633829	LC633823
	COS-AC24032021	Mueang, Amnat Charoen				
	COS-UR24032021	Det Udom, Ubon Ratchathani				
	COS-NS28032021	Mueang, Nakhon Sawan				
	COS-PC30032021	Wachirabarami, Phichit				
	COS-PB08042021	Lom Sak, Phetchabun				
	Crude drugs					
	LPC-NP08082018	Mueang, Nakhon Pathom	-	-	-	-
*Morinda citrifolia* L.	Authentic specimens					
	MOC-PL08032021	Mueang, Phitsanulok				
	MOC-KP06032021	Mueang, Kamphaeng Phet				
	MOC-CM11032021	Mueang, Chiang Mai	LC633821	LC633827	LC633830	LC633824
	MOC-AC24032021	Mueang, Amnat Charoen				
	MOC-UR24032021	Det Udom, Ubon Ratchathani				
	MOC-PL29032021	Phrom Phiram, Phitsanulok				
	Crude drugs					
	YOC-CM07082019	Mueang, Chiang Mai	-	-	-	-

**Table 3:** Sequence Analysis and Pairwise Distance of *Aegle marmelos* (L.) Corrêa (AEM), *Coriandrum sativum* L. (COS), and *Morinda citrifolia* L. (MOC).

Regions	ITS2			ITS			*mat*K			*psb*A-*trn*H			*rbc*L		
Species	AEM	COS	MOC	AEM	COS	MOC	AEM	COS	MOC	AEM	COS	MOC	AEM	COS	MOC
Length (bp)	230	221	212	630	598	566	779	845	837	460	240	365	750	750	751
%GC Content	71.7	55.7	71.2	63.8	55.7	64.5	35.6	35.9	34.1	29.3	33.8	24.7	44.9	43.7	42.9
Pairwise Distance															
AEM	0	0.070	0.057	0	0.037	0.034	0	0.022	0.023	0	0.061	0.078	0	0.012	0.013
COS	0.536	0	0.087	0.423	0	0.032	0.263	0	0.020	0.455	0	0.065	0.087	0	0.010
MOC	0.401	0.628	0	0.383	0.336	0	0.282	0.243	0	0.681	0.472	0	0.111	0.075	0
Overall Mean Distance (S.E.)	0.522 (0.06)			0.381 (0.03)			0.263 (0.02)			0.536 (0.05)			0.091 (0.01)		

**Table 4:** List of plants used in TS recipe.

	No.	Scientific Name	Family	Part Used
TS Recipe	1.	*Aegle marmelos* (L.) Corrêa	Rutaceae	Fruit
	2.	*Coriandrum sativum* L.	Apiaceae	Fruit
	3.	*Morinda citrifoli* L.	Rubiaceae	Fruit

**Table 5:** Lists of Primers used in this study.

Primers	Sequences 5′ → 3′	Tm (°C)	Reference
ITS2 intergenic region			
Un.3F	CGA CTC TCG GCA AGG GAT AT	56.73	[57]
Akebi-26SR	GTA AGT TTC TTC TCC TCC GC	52.89	[58]
Internal transcribed spacer (ITS)			
ITS5A	CCT TAT CAT TTA GAG GAA GGA G	49.22	[59]
ITS4	TCC TCC GCT TAT TGA TAT GC	51.67	[60]
maturase K (*mat*K)			
matK-1RKIM-f	ACC CAG TCC ATC TGG AAA TCT TGG TTC	60.74	[61]
matK-3FKIM-r	CGT ACA GTA CTT TTG TGT TTA CGA G	53.29	
*trn*H-*psb*A intergenic spacer			
trnH-psbAF *	ACT GCC TTG ATC CAC TTG GC	58.31	[62]
trnH-psbAR *	CGA AGC TCC ATC TAC AAA TGG	53.32	
MOC-psbAF **	GCGCATGATGGATTCACAAT	53.50	This study
MOC-psbAR **	GAAGTTATGCACGAACGTAAT	50.01	
ribulose-1,5-bisphosphate carboxylase/oxygenase large subunit (*rbc*L)			
rbcL 1F	ATC TCA CCA CAA ACA GAA AC	50.22	[63]
rbcL 724R	TCG CAT GTA CCT GCA GTA GC	57.64	

* to use for Aegle marmelos and Coriandrum sativum; ** to use for Morinda citrifolia.

## Data Availability

Not applicable.

## Supplementary Materials

The following are available online at https://www.mdpi.com/article/10.3390/plants10102023/s1: Alignment of nucleotide sequences of ITS, S2: Alignment of nucleotide sequences of ITS2, S3: Alignment of nucleotide sequences of *mat*K, S4: Alignment of nucleotide sequences of *rbc*L, S5: Alignment of nucleotide sequences of *trn*H-*psb*A, S6: Microscopic character of *Aegle mamelos* (L.) Corrêa, *Coriandrum sativum* L., *Morinda citrifolia* L. fruit powder and TS powder.
