# Supplementary material for: Specification and DNA Barcoding of Thai Traditional Remedy for Chronic Kidney Disease: Pikad Tri-phol-sa-mut-than
Source: Plants (Basel). 2021 Sep 26;10(10):2023. doi: 10.3390/plants10102023 (PMC8540904; doi:10.3390/plants10102023)
Supplement: Supplementary file 1 [file plants-10-02023-s001.zip › plants-1325492-supplementary.pdf]

Article

Specification and DNA Barcoding of Thai Traditional Remedy for Chronic Kidney Disease: Pikad Tri-phol-sa-mut-than

Suwimol Thariwong [1,2], Aekkhaluck Intharuksa [1], Panee Sirisa-ard [3], Wannaree Charoensup [1] and Sunee Chansakaow [1,*]

[1] Department of Pharmaceutical Sciences, Faculty of Pharmacy, Chiang Mai University, Chiang Mai 50200, Thailand; suwimol.t@psru.ac.th (S.T.); identity.int@gmail.com (A.I.); wannareecharoensup@gmail.com (W.C.)

[2] Department of Integrated of Health, Aesthetics and Spa, Faculty of Science and Technology, Pibulsongkram Rajabhat University, Phitsanulok 65000, Thailand

[3] Faculty of Pharmacy, Chiang Mai University, Chiang Mai 50200, Thailand; pmpti008@gmail.com

* Correspondence: chsunee@gmail.com; Tel.: +66 5394 4342

Supplementary data

Figure S1 Alignment of nucleotide sequences of ITS

Figure S2 Alignment of nucleotide sequences of ITS2

Figure S3 Alignment of nucleotide sequences of *mat*K

Figure S4 Alignment of nucleotide sequences of *rbc*L

Figure S5 Alignment of nucleotide sequences of *trn*H-*psb*A

Table S1 Microscopic character of *Aegle mamelos* (L.) Corrêa, *Coriandrum sativum* L., *Morinda citrifolia* L. fruit powder and TS powder

```
             ....|....| ....|....| ....|....| ....|....| ....|....|
                     10         20         30         40         50
AEM-ITS      TCGAAACCTG CC-CGGCAGA ACGACCCGCG AACCAGTACA GAACACCGGC
COS_ITS      TCGAAACCTG CAGAAGCAGA ACGACCTGCT AACTCGTA-- -AACACATTG
MOC-ITS      TCGAATCCTG AC-CGGCAGA -----CCGCG AACTCGTTGA GCAAACTTCG

             ....|....| ....|....| ....|....| ....|....| ....|....|
                     60         70         80         90        100
AEM-ITS      GGCGGGAGGG GGGACGCGCT CCGCCGCGGG CGCACCTCCC TCCCCCCCGC
COS_ITS      GGCAAGCGTC GGG------- -------GGG CTTTTGTCCC TTGTTCGCGA
MOC-ITS      GGGTGCTGGC GGG------- ---------- ---------- ----------

             ....|....| ....|....| ....|....| ....|....| ....|....|
                    110        120        130        140        150
AEM-ITS      GCCGCGCGGA GGGGGACCCG TCCCGCTCCC CGCCGGCAAA ACAACGAACC
COS_ITS      ATCCCTGGTA GGTGGCCCCT CCTGGGTGGC CGCTGGCCTC AAAATCA--T
MOC-ITS      ------CGAG GGAAACCCCG CCCGTCCGCG CACCATCCCA ACTA--AACT

             ....|....| ....|....| ....|....| ....|....| ....|....|
                    160        170        180        190        200
AEM-ITS      CCCGGCGCGG TCCGCGCCAA GGAAAATGTA ACGAGAGAGC --ACGCTCCC
COS_ITS      TCGGGCGCGG AATGCGCCAA GGAACTTGAA ATTGAATTGT --ACGTCCGC
MOC-ITS      CTCGGCGCGG GACGCGCCAA GGACTACTCA AACGGATCGC CGGCCTCCCC

             ....|....| ....|....| ....|....| ....|....| ....|....|
                    210        220        230        240        250
AEM-ITS      GCGGCCCCGG AGACGGTGCG CCGCGGGGCG CGGCGCCTTC TTTCACGAGT
COS_ITS      ATCCCGT--- ---------- TAGCGGGCAG CGGCGTCATT CCA-------
MOC-ITS      GCGGCTT--- ---------- CCGCGGGGCG AGCTGCGCGT CTGGTCGTTT

             ....|....| ....|....| ....|....| ....|....| ....|....|
                    260        270        280        290        300
AEM-ITS      ATCCAAAACG ACTCTCGGCA ACGGATATCT CGGCTCTCGC ATCGATGAAG
COS_ITS      AAAAACAACG ACTCTCGACA ACGGATATCT CGGCTCTCGC ATCGATGAAG
MOC-ITS      AACTAAAACG ACTCTCGGCA ACGGATATCT AGGCTCTCGC ATCGATGAAG

             ....|....| ....|....| ....|....| ....|....| ....|....|
                    310        320        330        340        350
AEM-ITS      AACGTAGCGA AATGCGATAC TTGGTGTGAA TTGCAGAATC CCGTGAACCA
COS_ITS      AACGTAGCGA AATGCGATAC TTGGTGTGAA TTGCAGAATC CCGTGAACCA
MOC-ITS      AACGTAGCGA AATGCGATAC TTGGTGTGAA TTGCAGAATC CCGTGAACCA

             ....|....| ....|....| ....|....| ....|....| ....|....|
                    360        370        380        390        400
AEM-ITS      TCGAGTCTTT GAACGCAAGT TGCGCCCCAA GCCGTTAGGC CGAGGGCACG
COS_ITS      TCGAGTCTTT GAACGCAAGT TGCGCCCGAA GCCACTAGGC CGAGGGCACG
MOC-ITS      TCGAGTTTTT GAACGCAAGT TGCGCCCGAA GCCATTAGGC TGAGGGCACG

             ....|....| ....|....| ....|....| ....|....| ....|....|
                    410        420        430        440        450
AEM-ITS      TCTGCCTGGG TGTCACGCAT CG--TTGCCC CACCCCTCCC CCTCCCCCGG
COS_ITS      TCTGCCTGGG TGTCACGCAT TGTCTTGCC- CACAACCACC CACTCCTTGA
MOC-ITS      TCTGCGTGGG CGTCACGCAT CGCGTCGCCA CCCCCCTCCT CGCCCCGCGC

             ....|....| ....|....| ....|....| ....|....| ....|....|
                    460        470        480        490        500
AEM-ITS      GGGAACGGAT GG---TGCGG GCGGAGATTG GCCTCCCGTC CGCCAACTGC
COS_ITS      GGAGTTGTGT TGGTTTGGGG GTGGAAACTG GCCTCCCGT- -GCC--TTGT
MOC-ITS      GGGAACGACG TG----GGTG GCGGATGTTG GCCGCCCGT- -GCCCTCCGC
```

```
                       510        520        530        540        550
AEM-ITS     GG-GCGGTTG GCCCAAATCC GAGTCCTCGG CGACCGAAGC CGCGGCGATC
COS_ITS     CGCGCGGTTG GCGGAAAATC GAGTCTTCGA CGACGGATGT CGTGAC-ATC
MOC-ITS     GGCGCGGCTG GCCTAAATGC GAGTCCTCGG CCCGGGACGT CACGGCGAGT

            ....|....| ....|....| ....|....| ....|....| ....|....|
                   560        570        580        590        600
AEM-ITS     GGTGGTGAAA CAAAAAAGCC TCTCGAGCTC CCGCCGCG-C GCCCGGTCTC
COS_ITS     GGTGGTTGTA --AAAGGCCC TCTTGTCTTG TCAC-GCGAA TCCTAGTCAT
MOC-ITS     GGTGGTTGAA CTCATCAA-C TCGAGAGCCG TCGCGACGAC GCCCGACG--

            ....|....| ....|....| ....|....| ....|....| ....|....|
                   610        620        630        640        650
AEM-ITS     CGCGTGGGGA CTCCGCGACC CTAGTG---- ----CCCCGC ACGAGCGGCG
COS_ITS     CTTA-GCGAG CTCCAGGACC CTTAGGCGCA CACACTCTGT GCG-------
MOC-ITS     -----GGGAA CTCTCCGACC TTGGAG---- ----CCCTTC GCGAGC----

            ....|....| ...
                   660
AEM-ITS     GCTCGCACGG CGA
COS_ITS     -------CTT TGA
MOC-ITS     -------CCT CGA
```

Figure S1. Alignment of nucleotide sequences of ITS.

```
            ....|....| ....|....| ....|....| ....|....| ....|....|
                    10         20         30         40         50
AEM-ITS2    CGCATCGTTG CCCCACCCC- -TCCCCCTCC CCC-GGGGGA ACGGATGG--
COS-ITS2    ATTGTC-TTG -CCCACAACC ACCCAC-TCC --TTGAGGAG TTGTGTTGGT
MOC-ITS2    ATCG-CGTCG --CCACCCCC CTCCTC-GCC CCGCGCGGGA ACGACGTG--

            ....|....| ....|....| ....|....| ....|....| ....|....|
                    60         70         80         90        100
AEM-ITS2    -TGCGGGCGG AGATTGGCCT CCCGTCCGCC GACTGCGG-G CGGTTGGCCC
COS-ITS2    TTGGGGGTGG AAACTGGCCT CCCGT--GCC --TTGTCGCG CGGTTGGCGG
MOC-ITS2    -GGTGG-CGG ATGTTGGCCG CCCGT--GCC CTCCGCGGCG CGGCTGGCCT

            ....|....| ....|....| ....|....| ....|....| ....|....|
                   110        120        130        140        150
AEM-ITS2    AAATCCGAGT CCTCGGCGAC CGAAGCCGCG GCGATCGGTG GTGAAACAAA
COS-ITS2    AAAATCGAGT CTTCGACGAC GGATGTCGTG AC-ATCGGTG GTTGTA-AAA
MOC-ITS2    AAATGCGAGT CCTCGGCCCG GGACGTCACG GCGAGTGGTG GTTGAACTCA

            ....|....| ....|....| ....|....| ....|....| ....|....|
                   160        170        180        190        200
AEM-ITS2    AAGCCTCTCG AGCTCCCGCC GCG-CGCCCG GTCTCCGCGT GGGGACTCCG
COS-ITS2    GGCCCTCTTG TCTTGTCAC- GCGAATCCTA GTCATCTT-A GCGAGCTCCA
MOC-ITS2    TCAACTCGAG AGCCGTCGCG ACGACGCCCG AC-------G GGGAACTCTC

            ....|....| ....|....| ....|....| ....|..
                   210        220        230
AEM-ITS2    CGACCCTAGT GCCCCGCACG AGCGGCGGCT CGCACGG
COS-ITS2    GGACCCTTAG GC---GCACA CACTCTGTGC GCTTTGA
MOC-ITS2    CGACCTTGGA GCCCTTCGCG AGC------- -CCTCGA
```

Figure S2. Alignment of nucleotide sequences of ITS2.

```
                ....|....| ....|....| ....|....| ....|....| ....|....|
                        10         20         30         40         50
AEM-matK        TCGAAC---- ---------- ---------- ---------- ----------
COS-matK        TCAAACTATT CGCTACTGGG TAAAAGACGC TTCTTCTTTA CATTTATTAA
MOC-matK        TCAAACCCTT CATTATTGGG TAAAAGATGC CTCCGCCTTG CATTTATTAC

                ....|....| ....|....| ....|....| ....|....| ....|....|
                        60         70         80         90        100
AEM-matK        ---------- ---------- ---AGTCTTA TTACTTCAAA GAACTCTATT
COS-matK        GATTCTTTCT CCACGAGTAT CGTATTTGTA ATACTCCAAA TAAAGCCACT
MOC-matK        GATTATTTTT CCACGAATAT TGGACTCTTA GTGCTACAAA GAAATCCCAT

                ....|....| ....|....| ....|....| ....|....| ....|....|
                       110        120        130        140        150
AEM-matK        TCTGTTTTTT TAAAAAGTAA TCCAAGATTG TTATTGTTTC TATATAATTC
COS-matK        TCTTGTTTTT CAAAAAGAAA TCGAAGGTTT TTCTTCGTCC TATACAATTC
MOC-matK        T---TTTCAC CAAAAAGAAA TAAAAGATTT TTTTTCTTAT TATATAATTC

                ....|....| ....|....| ....|....| ....|....| ....|....|
                       160        170        180        190        200
AEM-matK        TCATGTATAT GAATATGAAT CCATCCTCTT TTTTCTCTGT AACCAATCGT
COS-matK        TCATCTATGT GAATACGAAT CCATCTTCGT CTTTCTTCGT AACAAATCTT
MOC-matK        TCATGTATAT GAATACGAAT CCATTTTGGC CTTTCTCCGT AACCAATCTT

                ....|....| ....|....| ....|....| ....|....| ....|....|
                       210        220        230        240        250
AEM-matK        CTCATTTACA ATCAACATCC TCTCGAGTCC TCGTTGAGCG AACGTATTTC
COS-matK        CTCATTTATG CTCAACGTCT TCTGGAACCC TTCTTGAACG AATCTTTTTC
MOC-matK        CGCATTTGCA ATCAACATCT TTTGTATTAT TTCTCGAACG ACTTTATTTT

                ....|....| ....|....| ....|....| ....|....| ....|....|
                       260        270        280        290        300
AEM-matK        TATGGAAAAG TCGAATAT-- ----CTTGTC GAAGTCTTTG CTAAAGATTT
COS-matK        TATGGAAAAC TAGAACATCT TGGACTTGTA GAAGCTTTTG CTAAGGCCGT
MOC-matK        TATGGAAAAA AAGAACGT-- ----CTTGTA GAAGTTGTTG CTAAGGATTT

                ....|....| ....|....| ....|....| ....|....| ....|....|
                       310        320        330        340        350
AEM-matK        TCAGGACATC TTAGGGTTGT TCAAGGATCC TTTCATGCAT TATGTTAGAT
COS-matK        TCAGGACAAT CTGTGGTTGT TTAAGGACCC TTTCATGCAT TATATTAGTT
MOC-matK        TCGGGTTAGT CTATGGCTGT TCACAGACCC TTTCATGCAG TATGTTAGGT

                ....|....| ....|....| ....|....| ....|....| ....|....|
                       360        370        380        390        400
AEM-matK        ATCAAGGAAA ATCCATTTTG GCTTCAAAGG ATACGCCTCT TCTGATGAAT
COS-matK        ATCAAGGAAA ATCAATTCTC GCTTCAAAAG GGACGCCCCT TTTGATGAAA
MOC-matK        ATCAAGGAAA ATCAATTCTG CTTTCAAAGG GTACACCTCT TTTGATGAAT

                ....|....| ....|....| ....|....| ....|....| ....|....|
                       410        420        430        440        450
AEM-matK        AAATGGAAAT ATTACCTTGT CGGTTTATGG CAATGGCATT TTCACGTGTC
COS-matK        AAATGGACAT ATTATTTTGT TAATTTATGG AAATGTCATT TTTACCTATG
MOC-matK        AAATGGAAAT CTTATCTTGT CAATTTTTGG CAATATCACT TTGATCTGTG

                ....|....| ....|....| ....|....| ....|....| ....|....|
                       460        470        480        490        500
AEM-matK        TTCTCAACCA GGAAGGGTTC AGCTAAACCA CTTATACTTA GAAAAGTACG
COS-matK        GTCTCAGCCG GGACGGATCT GTATAAACCA ATTATAT--- ---AATCATT
MOC-matK        GTTTCGCTCG GGAAGGGTTT ATATAAATCA ATTTTCT--- ---AATCATT
```

```
             ....|....| ....|....| ....|....| ....|....| ....|....|
                    510        520        530        540        550
AEM-matK     CTATTAACTT TCTGGGCTAT CTTTCCGGTG TGCGACTAAA TTCTTTGTTG
COS-matK     CCCTAGCTCT TCTGGGCTAT CTATCAAGTG CGCGACTAAA CCCTTCAATG
MOC-matK     CACTTGACTT TGTGGGCTAT CGTTCAAGTG TGCGACTAAA TCCGGCAATG

             ....|....| ....|....| ....|....| ....|....| ....|....|
                    560        570        580        590        600
AEM-matK     GTACGAAGTC AAATGCTAGA AAATTCATTT CTAATAGGTA ATTCTATGAA
COS-matK     GTACGCAGTC AAATGCTAGA AAATGCATTT ATAATTGATA ATCCTATTAA
MOC-matK     GTACGGGGTC AAATGCTAGA AAATTCATTT CTAATTAATA ATGCTATTAA

             ....|....| ....|....| ....|....| ....|....| ....|....|
                    610        620        630        640        650
AEM-matK     GAAGGTCGAT ACCACCGTTC CAATTATTCA TCTGATTGGA TCATTGACTA
COS-matK     TAAGTTCGAT ACTCTTGTTC CAATTGTTCC TCTAATTGGA TCATTGGCTA
MOC-matK     GAAATTGGAT ATGCTTGTTC CAATTATTCC TCTTATTAGA TCATTGGCTA

             ....|....| ....|....| ....|....| ....|....| ....|....|
                    660        670        680        690        700
AEM-matK     AGGCGAGGTT TTGTAACGTA TTAGGGCATC CTATCAGTAA GTCGACTTGG
COS-matK     AGGCGAGATT TTGTAACGTA TTGGGGCACC CTATTAGTAA GGCGGTTTGG
MOC-matK     AAGCGAAATT TTGTAACCTA TTAGGACATC CCCTTAGTAA GCCGGTTTGG

             ....|....| ....|....| ....|....| ....|....| ....|....|
                    710        720        730        740        750
AEM-matK     GCCGATTTAT CTGATTCTCA TCTTATCGAC CGATTTGTGC GTATATGCAG
COS-matK     ACTGATTTAT CAGATTCTGA TATTGTTGTC CGATTTGGGC GTATCTGCAG
MOC-matK     ACTGATTTAT CAGATTCTGA TATGATTGAA CGCTTTGGGT ATATATGCAG

             ....|....| ....|....| ....|....| ....|....| ....|....|
                    760        770        780        790        800
AEM-matK     AAATCTTTCT CATTATTACA GCGGATCTTC AAAAAAAAAA AGTTTGTATC
COS-matK     AAATATTTAT CATTATTATA GTGGATCCTC ACAAAAAAAA AGTTTGTATC
MOC-matK     AAACCTTTCT CATTATCATA GCGGGTCTTC AAAAAAAAAG ATTTTGTATC

             ....|....| ....|....| ....|....| ....|....| ....|....|
                    810        820        830        840        850
AEM-matK     GAGTAAAATA TATACTTCGG CTTTCTTGTG TTAAAAGTTT GGTTCGAAAC
COS-matK     GAATAAAGTA TATACTTCGA CTGTCTTGTG CTAGAACTTT GGCTCGTAA-
MOC-matK     GAATAAAGTA TATACTTCGA CTTTCTTGTG CTAAAACTTT GGCGGGTAAA

             ..

AEM-matK     CA
COS-matK     CA
MOC-matK     CA
```

Figure S3. Alignment of nucleotide sequences of *mat*K.

```
                ....|....| ....|....| ....|....| ....|....| ....|....|
                        10         20         30         40         50
AEM-rbcL        TTCTATTATG TCACCC-CCA ACAGAAACTA AAGCGAGTGT TGGATTCAAG
COS-rbcL        TTCTATTATG TCACCCACAA ACAGAAACTA AAGCAGGTGT TGGATTCAAA
MOC-rbcL        TTCTATTATG TCACCCACAA ACAGAAACTA AAGCAAGTGT TGGATTCAAA

                ....|....| ....|....| ....|....| ....|....| ....|....|
                        60         70         80         90        100
AEM-rbcL        GCCGGTGTTA AAGATTATAA ATTGACTTAT TATACTCCTG ACTATGTAAC
COS-rbcL        GCTGGGGTTA AAGATTACAA ATTGACTTAT TATACTCCTG ACTATGAAAC
MOC-rbcL        GCTGGTGTTA AAGAGTACAA ATTGACTTAT TATACTCCTG AATACGAAAC

                ....|....| ....|....| ....|....| ....|....| ....|....|
                       110        120        130        140        150
AEM-rbcL        CAAAGATACT GATATCTTGG CAGCATTCCG AGTAACTCCT CAGCCCGGAG
COS-rbcL        CAAAGATACT GATATCTTGG CAGCATTCCG AGTAACTCCT CAACCTGGAG
MOC-rbcL        CAAAGATACT GATATCTTGG CAGCATTCCG AGTAACTCCC CAACCTGGAG

                ....|....| ....|....| ....|....| ....|....| ....|....|
                       160        170        180        190        200
AEM-rbcL        TTCCACCCGA GGAAGCGGGG GCTGCGGTAG CTGCGGAATC CTCTACTGGT
COS-rbcL        TTCCACCTGA AGAAGCGGGG GCCGCGGTAG CTGCCGAATC TTCTACTGGT
MOC-rbcL        TTCCACCGGA AGAAGCAGGG GCCGCGGTAG CTGCCGAGTC TTCTACTGGT

                ....|....| ....|....| ....|....| ....|....| ....|....|
                       210        220        230        240        250
AEM-rbcL        ACCTGGACAA CTGTGTGGAC CGATGGGCTT ACCAGCCTTG ATCGTTACAA
COS-rbcL        ACATGGACCA CTGTGTGGAC CGATGGACTT ACCAGCCTTG ATCGTTACAA
MOC-rbcL        ACATGGACAA CTGTATGGAC GGATGGACTT ACCAGTCTTG ATCGTTACAA

                ....|....| ....|....| ....|....| ....|....| ....|....|
                       260        270        280        290        300
AEM-rbcL        AGGGCGATGC TACAACATTG AGCCCGTTGC TGGAGAAGAG AATCAATATA
COS-rbcL        AGGGCGCTGC TACGAAATCG AGCCCGTTGC TGGAGAAGAA AATCAATATA
MOC-rbcL        AGGGCGATGC TACCACATCG AGCCAGTTCC TGGAGAAGAA GATCAATATA

                ....|....| ....|....| ....|....| ....|....| ....|....|
                       310        320        330        340        350
AEM-rbcL        TATGTTATGT AGCTTACCCG TTAGACCTTT TTGAAGAAGG TTCTGTTACT
COS-rbcL        TCGCTTATGT AGCTTACCCA TTAGACCTTT TTGAAGAAGG TTCTGTTACT
MOC-rbcL        TTGCTTATGT AGCTTACCCG TTAGACCTTT TTGAGGAAGG TTCTGTTACT

                ....|....| ....|....| ....|....| ....|....| ....|....|
                       360        370        380        390        400
AEM-rbcL        AACATGTTTA CTTCCATTGT GGGTAATGTA TTTGGTTTCA AAGCACTGCG
COS-rbcL        AACATGTTTA CTTCCATTGT AGGTAATGTA TTTGGGTTCA AAGCCCTGCG
MOC-rbcL        AACATGTTTA CTTCCATTGT AGGTAATGTA TTTGGGTTCA AAGCCCTGCG

                ....|....| ....|....| ....|....| ....|....| ....|....|
                       410        420        430        440        450
AEM-rbcL        CGCTCTACGT CTAGAGGATC TACGAATCCC TCCTGCGTAT TCTAAAACTT
COS-rbcL        CGCTCTACGT CTGGAAGATC TGCGAATCCC CGTTGCTTAT GTTAAAACTT
MOC-rbcL        CGCTCTGCGT CTGGAAGATT TGCGAGTTCC CATTTCTTAT ATTAAAACCT
```

```
AEM-rbcL      TCCAAGGCCC GCCTCACGGC ATCCAAGTTG AGAGAGATAA ATTGAACAAG
COS-rbcL      TCCAAGGACC GCCTCATGGC ATCCAAGTTG AGAGAGATAA ATTGAACAAA
MOC-rbcL      TCCAAGGCCC GCCTCATGGC ATTCAAGTCG AGAGAGATAA ATTGAACAAG

              ....|....| ....|....| ....|....| ....|....| ....|....|
                     510        520        530        540        550
AEM-rbcL      TATGGCCGTC CCCTGTTGGG ATGTACTATT AAACCTAAAC TGGGGTTATC
COS-rbcL      TATGGTCGTC CCCTGTTGGG ATGTACTATT AAACCTAAAT TGGGGTTATC
MOC-rbcL      TATGGTCGTC CCCTGTTGGG ATGTACTATT AAACCGAAAT TAGGTTTATC

              ....|....| ....|....| ....|....| ....|....| ....|....|
                     560        570        580        590        600
AEM-rbcL      CGCTAAGAAT TATGGTAGGG CGGTTTATGA ATGTCTACGT GGTGGACTTG
COS-rbcL      CGCTAAAAAC TACGGTAGAG CGGTTTATGA ATGTCTCCGC GGTGGACTTG
MOC-rbcL      TGCTAAAAAC TATGGTAGAG CAGTTTATGA ATGTCTTCGT GGTGGACTTG

              ....|....| ....|....| ....|....| ....|....| ....|....|
                     610        620        630        640        650
AEM-rbcL      ACTTTACCAA AGATGATGAG AACGTGAACT CCCAACCATT TATGCGTTGG
COS-rbcL      ATTTTACCAA AGACGATGAG AATGTGAACT CCCAACCATT TATGCGTTGG
MOC-rbcL      ATTTTACCAA AGATGATGAA AACGTGAACT CCCAACCATT TATGCGTTGG

              ....|....| ....|....| ....|....| ....|....| ....|....|
                     660        670        680        690        700
AEM-rbcL      AGGGACCGTT TCTTATTTTG TGCGGAAGCA CTTTATAAAG CGCAAGCTGA
COS-rbcL      AGAGATCGTT TCTTATTTTG TGCCGAAGCA ATTTATAAAG CACAGGCTGA
MOC-rbcL      AGAGATCGTT TCTTATTTTG TGCTGAAGCC CTTTTTAAAG CGCAGTCCGA

              ....|....| ....|....| ....|....| ....|....| ....|....|
                     710        720        730        740        750
AEM-rbcL      AACAGGTGAA ATCAAAGGTC ATTACTTGAA TGCTACTGCA GGTACATGCG
COS-rbcL      AACTGGTGAA ATCAAAGGGC ATTACTTGAA TGCTACTGCA GGTACATGCG
MOC-rbcL      AACAGGTGAA ATCAAAGGGC ATTACTTGAA TGCTACTGCA GGTACATGCG

              .

AEM-rbcL      A
COS-rbcL      A
MOC-rbcL      A
```

Figure S4. Alignment of nucleotide sequences of *rbc*L.

```
               ....|....| ....|....| ....|....| ....|....| ....|....|
                      10         20         30         40         50
AEM-psbA       CAAATTCAAC CATTGATCAT TTTTTTTTTA TCTTATCTTA CTTATGAAAA
COS-psbA       CCAGTTACTG CCTTGATCCA CTTGGCTACA TCCGCCCC-- ----------
MOC-psbA       CAATCCACTG CCTTAATCCA CTTGGCCACA TCCGCCCCCT ATT-------

               ....|....| ....|....| ....|....| ....|....| ....|....|
                      60         70         80         90        100
AEM-psbA       GCCAAATGAA GATCGAAGGG CAGAAAACTA CAACCTTTCT ATTGTCTTTT
COS-psbA       GCCAA----- ---------- ---------- ------TTTT ATTTTATTTA
MOC-psbA       -CTAA----- ---------- ---------- -----TTTTT AATGT-TTCA

               ....|....| ....|....| ....|....| ....|....| ....|....|
                     110        120        130        140        150
AEM-psbA       TTCTTTGCTA TGAAATTAAC TGTAAAATTC GAAAATGCGA CTCTAG----
COS-psbA       TTTTT----- ---------- ------ATTT CAAAGGA--- ----------
MOC-psbA       TTCAT----- ---------- ---AAAATTC TATAATTCTA TTTTGAACAT

               ....|....| ....|....| ....|....| ....|....| ....|....|
                     160        170        180        190        200
AEM-psbA       --TTTCTAAT TAATAATCTA ATTAAGTAAA ATTAACTCAA TTCGAAATTT
COS-psbA       --TTCCTTTT TGATCATTCA -------AAA AT-------- ------ATTT
MOC-psbA       TTTACTTTCT TTAAACTTTA -------AAA ATTCTTAAAA TCTAGAATTC

               ....|....| ....|....| ....|....| ....|....| ....|....|
                     210        220        230        240        250
AEM-psbA       ATCAATTTAT TAGTAGTATT AGCGCATACC AAAAATATCA TACTAAATCA
COS-psbA       GTTTATCTA- ---------- ---------- ---------- ----------
MOC-psbA       TCTATTTTA- ---------- --GGTACGCC ---------- ----------

               ....|....| ....|....| ....|....| ....|....| ....|....|
                     260        270        280        290        300
AEM-psbA       AAGAAAAGAA AGAAAAAGTA TAAAAATACT TAAAAAAAAA AAAACGAAAA
COS-psbA       ---------- --AAACAGTC TGAAA----- TATAAAAAAA AG--------
MOC-psbA       ---------- --TAGTAGTA TATAA----- TGTAAAAAAA AAATAGAAAT

               ....|....| ....|....| ....|....| ....|....| ....|....|
                     310        320        330        340        350
AEM-psbA       AAAAAAAGAA TGAACTAAAA AACTATTAAA GAACCCCGAT AAAG--AAAC
COS-psbA       ---------- ---------- ---------- ---------- ----------
MOC-psbA       TATAAATGAA ATCATTAGAA ATTCATTTTC AATTTGAATT AAATTCAAAT

               ....|....| ....|....| ....|....| ....|....| ....|....|
                     360        370        380        390        400
AEM-psbA       CCGACTAAAT AACGGATCAA TACTGACCCC CCG-----GC TGGGGGTCAG
COS-psbA       ---------- -------CAA TACCACCCTC TTGATAGAAC AAGAGGGTGG
MOC-psbA       TGAAATACTA AACGAAATAA AATTAAAATA AAGTAGCAAT AAGAGGTCCT

               ....|....| ....|....| ....|....| ....|....| ....|....|
                     410        420        430        440        450
AEM-psbA       TATTGATCCG TTA------T TTTCAAAACC CCGCCTACAC AAAGACCAAA
COS-psbA       TATTGCTTTT TTT-----AT TTCAAAAAAC TCGTATATAC TAAAACCCG-
MOC-psbA       TATTGCTACT TTATTTTCTT TTTCAAAAAC TCCTATACAA TAACACGAAT

               ....|....| ....|....| ....|....
                     460        470
AEM-psbA       ATCTTATCCA TTTGTAGATG GAGCTTCGA
COS-psbA       GCCTTACCCA TTTGTAGATG GAGCTTCGA
MOC-psbA       GTGTTATCCA TTTATAGATG GGACATCTA
```

Figure S5. Alignment of nucleotide sequences of *trn*H-*psb*A.

Table S1. Microscopic character of *Aegle mamelos* (L.) Corrêa, *Coriandrum sativum* L., *Morinda citrifolia* L. fruit powder and TS powder.

Microscopics characters	*Aegle mamelos* (L.) Corrêa	*Coriandrum sativum* L.	*Morinda citrifolia* L.	TS powder
1. Epicarp tissue	+	+	+	+
2. Thick walled parenchyma	+++		+++	+++
3. Oil gland	+			
4. Prismatic crystals line	+			
5. Sclereids and stone cell	+++			++
6. Group of elongated sclereids	++			+
7. Vascular tissue	++	+	+	++
8. Seed hair	++			+
9. Cotyledon	+			
10. Bi-layer fusiform sclereids		+++		++
11. Mono-layer elongated faintly lignified sclereids of endocarp		++		+
12. Fragment of vitta		+		+
13. Testa		+		
14. Seed in sectional view		+++	+	++
15. Microspheroidal crystals of calcium oxalate		++	+	+
16. Thin walled parenchyma			+++	++
17. Acicular crystals			++	+
18. Yellowish fibrous layer of seed coat			+++	++
19. Resin masses			+++	+
20. Oil globules	++	+++	+	++
